# Nursing Problems and Their Solution

**Published:** 1920-10-02

**Authors:** 


### Nursing Problems and their Solution.

THE General Nursing Council had several acute problems brought to their notice at the first meeting of the autumn session, and the substantial unanimity which prevailed in dealing with them is an augury for good. A request from Dr. Addison that the Council would consider the minimum age for probationers in Poor-law infirmaries led to a highly interesting discussion on this point, and general satisfaction will be caused by the conclusion arrived at. The Council recommends the age of nineteen as that at which probationers should be received in Poor-law infirmaries instead of, as at present, twenty-one. The voluntary hospitals have, of course, always been at liberty to lower the age, and many have been in the habit of doing so. Many matrons agree with Miss Dowbiggin, who considers that at nineteen a nurse is easier to teach and more adaptable to rules than at twenty-one, and is more responsive to training. This view was supported by Sir Jenner Verrall. There is a widespread belief that by lowering the age many promising girls may be secured for service as nurses who would otherwise drift into other callings. Moreover, the effect will be to extend the best-working years of the nursing sister, a matter in itself of high moment to the profession.

The difficulties, experienced by small and special hospitals in securing probationers were brought to the notice of the Council by the Boards of nine institutions, which are unable to offer a full certificate to their probationers, and in consequence experience great difficulty in obtaining trained sisters. The small and special institutions press for a scheme for reduced training in large hospitals for the probationers who have gone through their prescribed course with credit. A recognised certificate of affiliation would meet the case, but the problem is by no means an easy one. The Chairman had the approval of all present in referring this matter to the Examination and Education Committee, and their report will be awaited with much interest. Round this point centres, in our judgment, the ultimate success of State registration. It is of enormous importance to the profession that a way shall be opened through which the partially-trained nurse may be encouraged to continue her training, for it is she who bars the way to universal registration.

Considerable disappointment will, we fear, be caused in V.A.D. circles by the definite rejection by the Council of their military service as a claim to State registration. On this point, however, there was no hesitation. It was unanimously agreed that a direct negative be returned to the letter from the Joint V.A.D. Committee inquiring whether the V.A.D. members who had served for three years during the war in a military hospital would be eligible for State registration. Nothing further can be done for them than the reduction of four years training to three, though Miss Yapp stated that at Ashton-under-Lyne some had been received as second-year nurses in the civilian wards.

